# Toll-Like Receptor 7 Activation Enhances CD8+ T Cell Effector Functions by Promoting Cellular Glycolysis

**DOI:** 10.3389/fimmu.2019.02191

**Published:** 2019-09-12

**Authors:** Qian Li, Yan Yan, Jia Liu, Xuan Huang, Xiaoyong Zhang, Carsten Kirschning, Haifeng C. Xu, Philipp A. Lang, Ulf Dittmer, Ejuan Zhang, Mengji Lu

**Affiliations:** ^1^Institute of Virology, University Hospital of Essen, University of Duisburg-Essen, Essen, Germany; ^2^Center of Clinical Laboratory, The Fifth People's Hospital of Wuxi, Affiliated Hospital of Jiangnan University, Wuxi, China; ^3^Department of Infectious Diseases, Union Hospital, Tongji Medical College, Huazhong University of Science and Technology, Wuhan, China; ^4^Hepatology Unit and Department of Infectious Diseases, Nanfang Hospital, Southern Medical University, Guangzhou, China; ^5^Institute of Medical Microbiology, University Hospital of Essen, University of Duisburg-Essen, Essen, Germany; ^6^Department of Molecular Medicine II, Medical Faculty, Heinrich-Heine-University, Düsseldorf, Germany; ^7^Mucosal Immunity Research Group, State Key Laboratory of Virology, Wuhan Institute of Virology, Chinese Academy of Sciences, Wuhan, China

**Keywords:** toll-like receptor 7, CD8+ T cells, PI3K-Akt-mTOR, glycolysis, IRF4

## Abstract

The activation of TLR7 signaling in T cells accelerates antigen-specific responses. Such responses play an essential role in eliminating viral infections and can be anti-tumorigenic. However, the underlying mechanisms of how TLR7 can promote the optimal function of CD8+ T cells remain unclear. To investigate how TLR signaling directly contributes to CD8+ T cell functions, we examine the activation of cellular TLR7-related pathways and functional and metabolic alterations in TLR7-stimulated T cells during T cell receptor (TCR) signaling. In the present study, we investigated the activation of CD8+ T cells in response to direct stimulation by TLR7 ligands. TLR7 stimulation could promote the effector functions of purified CD8+ T cells *in vitro*. The TLR7-induced activation of CD8+ T cells occurs if CD8+ T cells were primed by αCD3 activation and increasingly expressed TLR7. MyD88 and AKT-mTOR signaling plays a critical role in TLR7-induced T cell activation. In addition to the upregulation of immune-related genes, metabolic alterations in CD8+ T cells, including the upregulation of glucose uptake and glycolysis, occurred by TLR7 stimulation. Glycolysis was found to be regulated by the AKT-mTOR pathway and a downstream transcription factor IRF4. Blocking glycolysis by either direct glucose deprivation or modulating the mTOR pathway and IRF4 expression was found to impair T cell activation and functions. Taken together, the activation of TLR7 signaling promotes the effector functions of CD8+ T cells by enhancing cellular glycolysis.

## Introduction

Toll molecules comprise a class of highly conserved molecules that plays a vital role in the immune surveillance of pathogen-associated molecular patterns and host defense against many pathogenic microorganisms (1, 2). Upon activation by their ligands, Toll-like receptors (TLRs) induce various cellular anti-viral responses including the release of inflammatory cytokines and the maturation of antigen-presenting cells (APCs) (3–5). Thus, these receptors act as a bridge between innate and adaptive immunity in the host (6). The TLR signaling pathway plays an important role in inducing a functional antiviral CD8+ T cell response, which is critical for viral control and clearance. Our previous study in a hepatitis B virus (HBV) mouse model demonstrated that the IL-1R/TLR signaling pathway is required for the generation of functional HBV-specific CD8+ T cells (7).

The regulation of TLRs in APCs, such as plasmacytoid dendritic cells, leads to the indirect activation of T and B lymphocytes (8). T cell immunity can also be modulated by the direct activation of TLRs on T lymphocytes. For example, TLR2 was shown to be expressed on activated and memory T cells (9, 10). Further, TLR2 ligands modulate murine CD4 and CD8+ T cell effector functions *in vitro* by increasing cytokine production upon either anti-CD3 (αCD3) antibody or T cell receptor (TCR) stimulation (11–13). TLR2-activated effector CD8+ T cells also enhance the therapeutic effects of specific CD8+ T cells in an *in vivo* tumor model (14). Since TCR activation increases TLR2 expression on T cells, the additional activation of this receptor reduces the TCR threshold required for T cell proliferation, differentiation, and cytokine production (15–17). In addition, TLR2 can enhance the mRNA stability of *IFN-*γ at low antigen levels (18). Thus, TLR2 acts like a co-stimulatory molecule that directly enhances TCR signal-induced T cell activation, function, and survival (19).

During infection, vaccination, or immunomodulatory therapies, TLR7 has also been shown to regulate the CD8+ T cell response *in vivo*. For example, TLR7 activation in the woodchuck and chimpanzee models of HBV infection enhances the function of intrahepatic T cells (20, 21). Further, the oral TLR7 agonist GS-9620 has been reported to promote HBV-specific CD8+ T cell responses in clinical trials. In addition, the increased expansion and activation of CD8+ T cells, rather than CD4+ T cells, was observed in TLR7-stimulated hepatic tissue (22). These findings fit the concept that TLRs are required for adaptive immune responses during viral infection and the TLR7 ligands can promote the adaptive immune response *in vivo*. One possible mechanism associated with this effect is that TLR7 promotes cross-presentation *via* APCs to enhance CD8+ T cell responses (23).

Recently, several *in vitro* studies have pointed out that TLR7 is a potential co-stimulator for CD8+ T cell activation and function. Song et al. found an increased expression of TLR7 in CD8+ T cells from HIV-1-infected individuals. *In vitro* stimulation with TLR7 agonist increased the expression of immune activation markers of CD8+ T cells (24). Salerno et al. also reported that murine CD8+ T cells can be stimulated by TLR7 ligands, resulting in rapid IFN-γ production (25). These results indicate that TLR7 could directly activate the CD8+ T cells and regulate their functions. However, the underlying mechanisms are still unclear. Geng et al. reported that MyD88 signaling enhances T cell functions by increasing activation of the mTOR pathway in an Akt and protein kinase C-dependent manner, suggesting a relationship between TLR2 stimulation and metabolic processes (26). It was also shown that the mTOR pathway regulates metabolic processes in immune cells, including the stimulation of glycolysis through transcription factors such as hypoxia-inducible factor 1α (HIF1α), MYC, and interferon regulatory factor 4 (IRF4), which enhances glucose import and the expression of glycolytic genes (27–32). However, whether TLR7 ligands contribute to the immune activation of CD8+ T cells through cellular metabolism needs to be investigated. In the current study, we addressed the questions of whether and how TLR7 ligand stimulation directly regulates the effector function of CD8+ T cells.

## Materials and Methods

### Mice

C57BL/6 wild type (WT) mice were purchased from Harlan Winkelmann Laboratories (Borchen, Germany). TRIF^−/−^, MyD88^−/−^, TRIF/MyD88^−/−^ mice were bred under specific pathogen-free conditions at the Institute of Virology of the University Hospital Essen. IRF4^−/−^ mice were bred in the animal facility of Heinrich Heine University, Düsseldorf, Germany. For assaying the antigen-specific CD8+ T cell activation, splenocytes from inbred female DbGagL TCR transgenic (tg) mice were used. The DbGagLTCR tg mice were on a C57BL/6 or B6.SJL (CD45.1 congenic) background and >90% of the CD8+ T cells contained a TCR specific for the DbGagL Friend virus (FV) epitope (FV-TCR CD8+ T cells) (33). DbGagLTCR tg mice were kept in the Animal Care Center, University of Duisburg-Essen. All mice were at 6–8 weeks of age. Handling of animals was conducted in accordance with the Guide for the Care and Use of Laboratory Animals and according to the approval by the district government of Düsseldorf, Germany.

### Isolation of Lymphocytes From the Spleen and Purification of CD8+ T Cells *in vitro*

The entire spleen was isolated from naïve mice and single cell suspensions of mouse splenocytes were obtained according to a previously described protocol (34). For murine CD8+ T cell purification, splenocytes derived from 4 to 5 mice were pooled and subjected to negative selection by MACS (CD8a+ T cell Isolation Kit; Miltenyi, Germany) according to the manufacturer's instructions. About 2–3 × 10^7^ purified CD8+ T cells were obtained from each preparation and treated as indicated in triplicates. Human CD8+ T cells were purified from PBMCs by negative selection (CD8a+ T cell Isolation Kit; Miltenyi, Germany).

### T Cell Culture and Activation *in vitro*

T cells were cultured in RPMI medium (no glucose) containing 10% dialyzed serum (Thermo Fisher, Germany), 1 mM sodium pyruvate, 2 mM L-glutamine, with or without 10 mM glucose. For murine T cell activation, 96-well flat-bottom tissue culture plates were pre-coated with αCD3 antibody (5.0 μg/mL; eBioscience, Frankfurt, Germany), and cells were cultured with or without R837 (1 μg/mL)/R848 (10 μg/mL; InvivoGen, San Diego, CA, USA) for 24–48 h (5 × 10^5^ cells/well). For some experiments, cells were treated with the indicated inhibitors such as 2-DG (1 mM; Sigma, Germany), rapamycin (2 μM), and Akti-1/2 (1 μM; Sigma, Germany). For human T cell activation, 96-well flat-bottom tissue culture plates were pre-coated with αCD3 antibody (5.0 μg/mL; eBioscience, Frankfurt, Germany), and human CD8+ T cells were cultured with or without R848 (1 μg/mL; InvivoGen, San Diego, CA, USA) for 24 h (5 × 10^5^ cells/well). For the indicated experiments, human CD8+ T cells were treated with 2-DG (10 mM; Sigma, Germany), rapamycin (10 μM), and Akti-1/2 (5 μM; Sigma, Germany). For all experiments, triplicate wells were performed under each condition.

### Cell Surface and Intracellular Staining

Generally, cell surface and intracellular staining of murine immune cells were performed as described previously (35).

Cells surface staining was performed by using reagents including αCD8 (clone 53-6.7; Biolegend, Germany), αCD69 (clone H1.2F3; Biolegend), αCD44 (clone IM7; Biolegend), αCD62L (clone MEL-14; Biolegend), αCD25 (clone PC61.5; Biolegend), and αGlut1 (Novus Biologicals, Germany).

For intracellular staining, cells were permeabilized by using a Cytofix/Cytoperm intracellular staining kit (BD Biosciences, Germany) and stained with antibodies including αIFN-γ (clone XMG1.2; eBioscience, Germany), αIL-2 (clone JES6-5H4, Biolegend), αTNF-α (clone MP6-XT22; Biolegend), αphospho-mTOR(clone MRRBY; eBioscience), and αAkt (clone 55; BD Bioscience).

Nuclear transcription factor staining was performed by using True-Nuclear™ Transcription Factor Buffer Set (Biolegend). Cells were then stained with αT-bet (clone 4B10; Biolegend), αEomes (clone Dan11mag; eBioscience), and αIRF4 (clone IRF4.3E4; Biolegend).

Stained cells were detected using an LSRII (BD Biosciences) or Navios Flow Cytometer (Beckman Coulter, Germany) and data were analyzed using FlowJo software (version 10).

Fluorescence minus one (FMO) control stains were used to determine background levels of staining (Figure S11).

### Metabolism Assays

Flow cytometry and real-time RT-PCR were used to analyze the expression of glucose transporter1 (Glut-1). Glycolysis-related markers including Glut-1, hexokinase 2 (HK2), and lactate dehydrogenase (LDH-α) were quantified by either real-time RT-PCR or western blotting. Glucose uptake was measured by detecting the mean fluorescence intensity of the glucose analog 2-NBDG (ThermoFisher, Germany) in cells. For 2-NBDG staining, glucose-free media were used to culture the cells for 30 min. Cells were then incubated at 37°C with 200 μM 2-NBDG in glucose-free media for 20 min before cell surface staining. Lactate production was measured with a specific enzyme assay using the Lactate colorimetric/fluorometric kit (Biovision, Germany).

### Real-Time RT-PCR and Western Blotting

Total cellular RNA was extracted from 0.5 to 1 × 10^6^ cells using TRIzol (Life Technologies; Darmstadt, Germany) (36). The primers were purchased from QIAGEN, including *Glut-1* (QT01044953; QIAGEN, Germany), *HK2* (QIAGEN; QT00155582), and *LDH*α (QIAGEN; QT02325414). Reverse transcription was performed by using the High Capacity cDNA Reverse Transcription Kit (Applied Biosystems, Germany). RNA quantitation was performed using SYBR green and a StepOne Plus RT-PCR system according to the manufacturer's instructions (QIAGEN, Hilden, Germany).

Western blot analysis was performed according to the established protocol (37). Whole-cell lysates were performed using cell lysis buffer (Thermo Fisher Scientific, Germany). Equal amounts of protein (100 μg) were separated by SDS-PAGE and transferred to polyvinylidene difluoride membranes (Millipore, Germany). After blocking the non-specific sites with 5% BSA, specific antibodies including phospho-Akt (Cell Signaling, Germany), phospho-mTOR (Cell Signaling, Germany) and HK2 (Cell Signaling, Germany) were incubated with membranes. Immunoreactive bands were then developed with an enhanced chemiluminescence system (GE Healthcare, Germany).

### Enzyme-Linked Immunosorbent Assay (ELISA)

Supernatants were collected from cultured cells. The cytokines secretion (IFN-γ, TNF-α, and IL-2) was measured by a specific ELISA kit (Biolegend) based on the manufacturer's instructions. The OD at 405 nm was then measured using a microplate reader (Bio-Rad Model 550, Germany).

### Statistical Analyses

Statistical analyses were performed using GraphPad Prism software version 6 (GraphPad Software Inc., San Diego, CA). Data between different groups were analyzed by a One-way ANOVA test. For the blockade experiments, data were analyzed using the Two-way ANOVA test. The *p* < 0.05 were considered significant. Significant differences between different groups are marked as follows: ^*^p < 0.05, ^**^p < 0.01, ^***^p < 0.001. All experiments are representative of three or two independent experiments.

## Results

### TLR7 Stimulation Directly Enhances the Effector Function of CD8+ T Cells

To initially assess the immunomodulatory properties of TLR7 on CD8+ T cells, splenocytes from naïve mice were stimulated with the TLR7 ligand resiquimod (R848) in the presence of an activating αCD3 antibody. The results indicated that R848 could potently elevate the frequency of CD44+, CD69+, and IFN-γ+ CD8+ T cells (Figure S1). In addition, an increase in the T cell functionality including enhanced CD25 expression on CD8+ T cells and the upregulation of IFN-γ secretion was also observed in FV-TCR CD8+ T cells after co-culture with peptide-loaded DCs in the presence of R848 (Figure S2).

**Figure 1:**
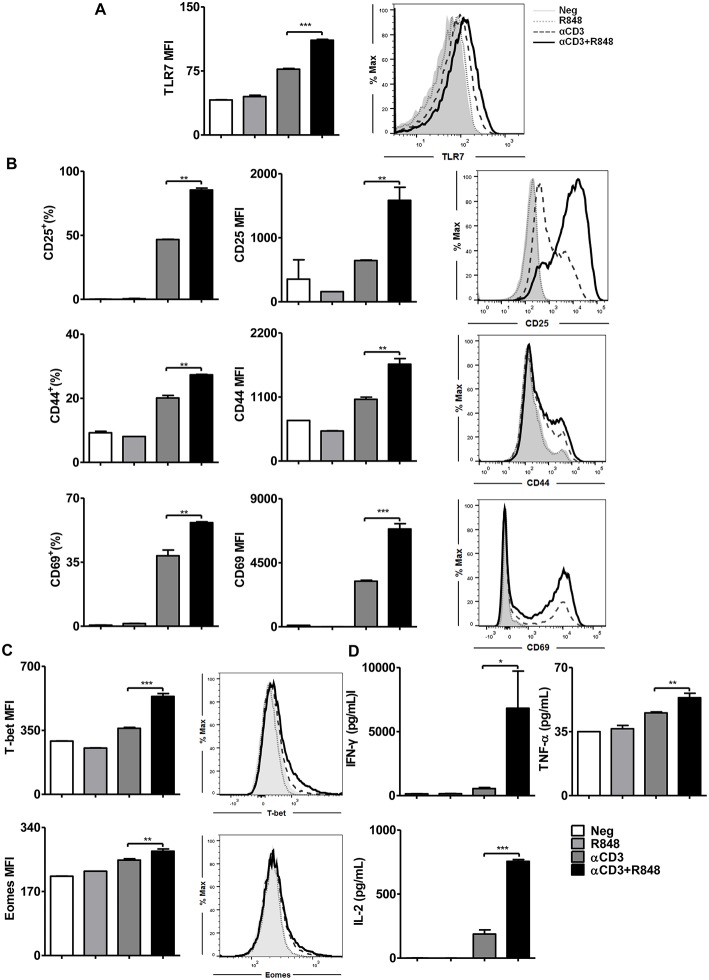
TLR7 ligands enhance CD8+ T cell activation and cytokine production. Purified CD8+ T cells were stimulated in plates with bound αCD3 antibody (5 μg/mL) alone or with R848 (10 μg/mL) for 24 h. TLR7 **(A)**, CD25, CD44, and CD69 **(B)**, and T-bet and Eomes **(C)** expression in CD8+ T cells were measured by flow cytometry. **(D)** Cytokines production including IFN-γ, TNF-α, and IL-2 was measured by specific ELISAs. Data are representative of three independent experiments. *N* = 3 wells per condition. All data are presented as mean ± SD. The statistical relevance was determined by One-way ANOVA: *p < 0.05; **p < 0.01; ***p < 0.001.

It has been reported that TLR7-activated APCs like plasmacytoid dendritic cells mediate cross-talk with CD8+ T cells (23). However, whether TLR7 ligands directly enhance the effector function of CD8+ T cells has not been examined to date. To test this, naïve splenic CD8+ T cells were highly purified from WT mice using magnet bead separation and then stimulated with an αCD3 antibody alone or in the presence of R848 for 24 h. Clearly, naïve CD8+ T cells could not be directly activated by TLR7 ligands unless they were stimulated synergistically with an αCD3 antibody (Figure 1A). This result was consistent with the fact that TLR7 is more expressed on activated T cells (38). Upon TLR7 stimulation, the expression of CD25, CD44, and CD69 was significantly increased (Figure 1B, Figure S3) as well as the expression of the transcription factors T-bet and Eomes (Figure 1C). In addition, the secretion of IFN-γ, TNF-α, and IL-2 by activated CD8+ T cells was augmented (Figure 1D). Imiquimod (R837), another TLR7 ligand, induced the maximum cell survival and cell activation at a dose of 1 μg/ml in the presence of αCD3 antibody compared to that with αCD3 antibody alone, whereas R848 at doses of 1–10 μg/ml appeared to improve cell viability and cell activation compared to that in the presence of αCD3 antibody only (Figure S4A). Moreover, the expression of the activation marker CD25 shows that R848 reached a plateau at a concentration as low as 0.1 μg/ml. Comparing the two TLR7 ligands at the optimal concentration for cell survival and activation, R848 (10 μg/ml) induced better effector functions in CD8+ T cells than R837 (1 μg/ml). Specifically, significantly enhanced CD25, CD44, and CD69 expression, as well as IFN-γ and TNF-α release by CD8+ T cells, was found with R848 co-stimulation compared to that with R837 (Figure S4B).

Thus, TLR7 serves as a co-stimulatory receptor for T cell activation in synergy with TCR signaling. Moreover, R848 was found to be a more efficient TLR7-activator for CD8+ T cell co-stimulation.

### Enhancement of CD8+ T Cell Effector Functions by R848 Is MyD88-Dependent

**Figure 2:**
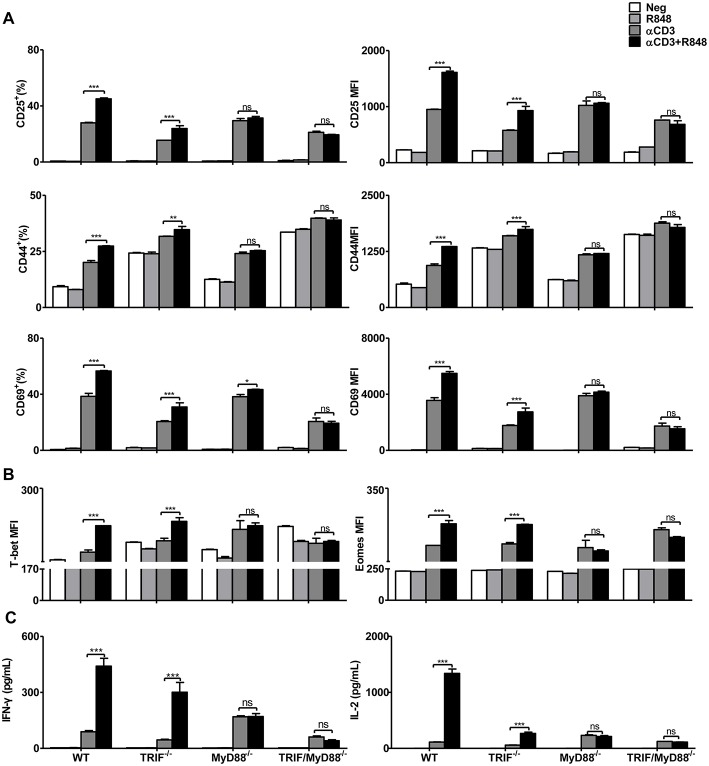
Enhancement of the effector function of CD8+ T cells mediated by TLR7 agonists is MyD88-dependent. Purified CD8+ T cells from WT, TRIF^−/−^, MyD88^−/−^, and TRIF/MyD88^−/−^ mice were stimulated in plates with bound αCD3 antibody (5 μg/mL) alone or with R848 (10 μg/mL) for 24 h. CD25, CD44, and CD69 **(A)**, T-bet and Eomes **(B)** expression in CD8+ T cells were analyzed by flow cytometry. **(C)** Cytokines production including IFN-γ and IL-2 was detected by specific ELISAs. Data are representative of two independent experiments. All data are presented as mean ± SD. The statistical relevance was determined by Two-way ANOVA: *p < 0.05; **p < 0.01; ***p < 0.001; ns, not significant.

Next, we investigated how TLR7 stimulation modulates the function of CD8+ T cells. It is known that intracellular TLR7 signaling occurs through the adaptor proteins MyD88 and TRIF (39). After stimulation with αCD3, CD8+ T cell activation, differentiation, and cytokine production were increased in WT, TRIF^−/−^, MyD88^−/−^, or TRIF/MyD88^−/−^ mice. CD25, CD44, and CD69 expression were further upregulated in WT and TRIF^−/−^ CD8+ T cells after αCD3 stimulation in the presence of R848 (Figure 2A). The transcription factors T-bet and Eomes were also significantly increased in CD8+ T cells upon stimulation with αCD3+R848 only in WT and TRIF^−/−^ CD8+ T cells (Figure 2B). Moreover, IFN-γ and IL-2 secretion was elevated in WT and TRIF^−/−^ CD8+ T cells under R848 co-stimulation (Figure 2C). However, R848 co-stimulation failed to further enhance the effector function of MyD88^−/−^ and TRIF/MyD88^−/−^ CD8+ T cells. MyD88 deficiency abolished R848-mediated enhanced CD8+ T cell effector function, confirming that this process is dependent on the MyD88 pathway.

Although the purity of isolated CD8+ T cells exceeded 96% (Figure S5), some residual non-CD8+ T cells were still present including APCs. Residual APCs in the fraction of purified CD8+ T cells may respond to R848, thus indirectly improving the functionality of CD8+ T cells. To exclude this possibility, MyD88^−/−^ splenocytes were mixed with WT splenocytes at a ratio of 1:1. Equal numbers of naïve WT and MyD88^−/−^ splenocytes were then subjected to CD8+ T cell purification by magnetic bead separation. MyD88^−/−^ CD8+ T cells were labeled with the indicated fluorescence marker CFSE and thereby distinguished from WT CD8+ T cells by flow cytometric analysis (Figure S6A). Purified WT and Myd88^−/−^ CD8+ T cells with some residual APCs were further stimulated with αCD3 with or without R848. Results showed that R848 increased CD69 expression, as well as IFN-γ production in WT CD8+ T cells but not in MyD88^−/−^ CD8+ T cells. Therefore, R848 acted directly on CD8+ T cells in the *in vitro* culture system (Figure S6B).

### mTOR Signaling Is Required for TLR7 Ligands to Regulate CD8+ T Cell Activation

**Figure 3:**
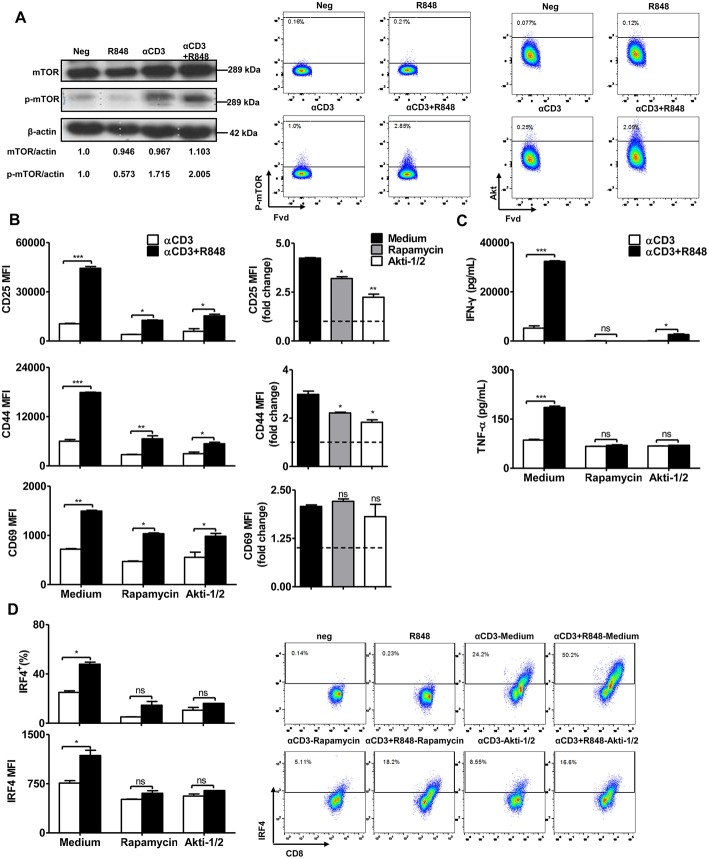
mTOR signaling regulates the effector function of CD8+ T cells. Purified CD8+ T cells were stimulated with αCD3 antibody (5 μg/mL) with or without R848 (10 μg/mL) for 48 h in the presence of rapamycin (2 μM) or Akti (1 μM). **(A)** mTOR and phosphorylated mTOR in CD8+ T cells were detected by western blotting. The level of phosphorylated mTOR and Akt were further determined by flow cytometry. **(B)** The activation of CD8+ T cells was assessed by staining with αCD25, αCD44, and αCD69 antibodies. The expression of CD25, CD44, and CD69 in the αCD3+R848 stimulated cells were expressed as fold changes compared to the αCD3 stimulated cells in the corresponding treatment of rapamycin/Akti-1/2. **(C)** IFN-γ and TNF-α secretion were detected by specific ELISAs. **(D)** IRF4 expression was shown by representative dot plots and MFI in CD8+ T cells. Data are representative of three independent experiments. Data are presented as mean ± SD. The statistical relevance was determined by One-way ANOVA (**B**, right panel) or Two-way ANOVA (**B**, left panel, **C,D**): *p < 0.05; **p < 0.01; ***p < 0.001; ns, not significant.

Previous studies have reported that the mTOR pathway is linked to MyD88 signaling during the engagement of TLRs in CD4 or CD8+ T cells (26, 40). To investigate whether TLR7 activation regulates mTOR signaling, we determined mTOR expression and phosphorylation by western blotting and flow cytometry. The results showed that αCD3 stimulation led to a high level of phosphorylated mTOR in CD8+ T cells. R848 further slightly increased the levels of mTOR, phosphorylated mTOR and Akt in CD8+ T cells (Figure 3A).

To confirm that mTOR signaling is required to regulate TLR7-mediated CD8+ T cell activation, CD8+ T cells were stimulated by αCD3 and/or R848 and treated with two different mTOR pathway inhibitors rapamycin and Akti-1/2. Results indicated that despite stimulation by the polyclonal αCD3 antibody, inhibition of mTOR pathway reduced the numbers of vital CD8+ T cells. However, we found that the numbers of vital CD8+ T cells were increased when R848 was co-applied in the presence of αCD3, and is dependent on the inhibition of the mTOR pathway (Figure S7A). Consistent with previous studies, inhibition of mTOR slightly reduced the αCD3-induced T cell activation, as indicated by a lower frequency of CD8+ T cells expressing activation markers, such as CD25 and CD44, and reduced production of IFN-γ by CD8+ T cells. In the presence of rapamycin, the use of R848 was still able to slightly upregulate the expression of CD25, CD44, and CD69. However, compared to the strong activation induced by R848 together with αCD3, the application of rapamycin or Akti-1/2 significantly suppressed the upregulation of CD25, CD44, and CD69 in CD8+ T cells by R848 treatment (Figure 3B, left panel, Figures S7B,C). The decreased expression of these activation markers in the αCD3+R848 stimulated cells, compared to the αCD3 only stimulated control, could be clearly judged in the format of fold changes (Figure 3B, right panel). Rapamycin and Akti-1/2 also abolished the further increase in IFN-γ and TNF-α production by CD8+ T cells in response to R848 (Figure 3C). The transcription factor IRF4 coordinates mTOR signaling to orchestrate immune activation and the function of T cells (41). We detected the expression of this marker by intracellular staining and found that enhanced IRF4 expression in response to R848 was abolished by rapamycin and Akti-1/2 treatment (Figure 3D). These results suggest that the blockade of Akt–mTOR pathway could significantly reduce or even abolish the R848-induced enhancement of T cell activation and function.

Taken together, these data show that the Akt–mTOR pathway is required for the TLR7-mediated enhancement of immune functions in CD8+ T cells.

### Akt-mTOR Signaling Pathway Plays an Important Role in TLR7-Mediated Improvement of Glycolysis in CD8+ T Cells

**Figure 4:**
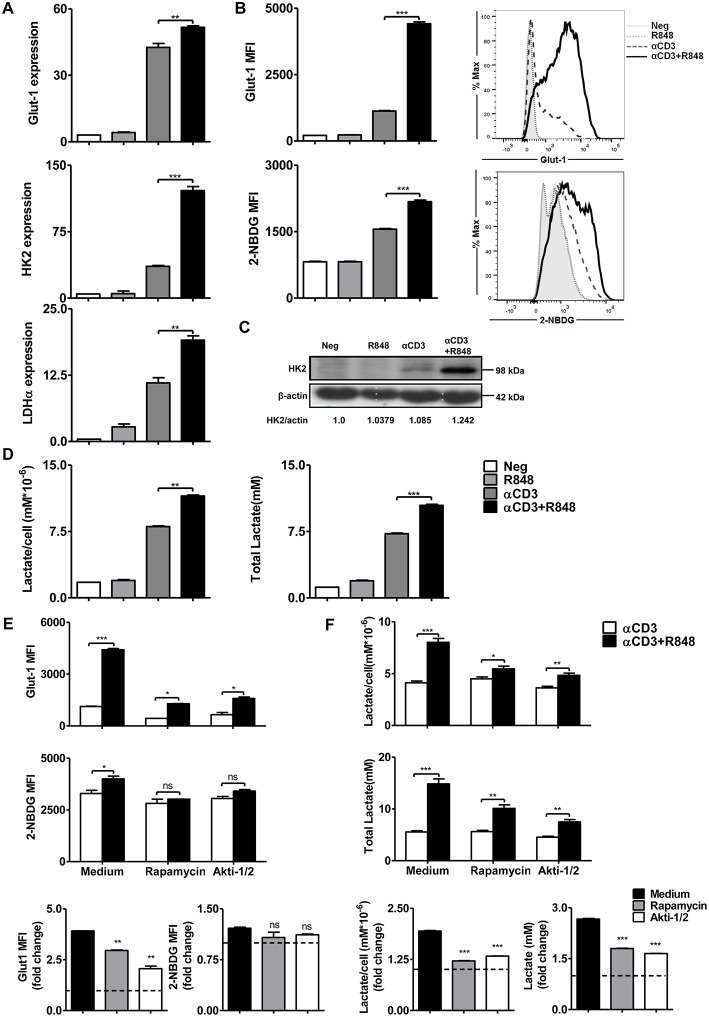
TLR7 agonists improve glycolytic metabolism *via* mTOR signaling in CD8+ T cells. Purified CD8+ T cells were stimulated with an αCD3 antibody (5 μg/mL) with or without R848 (10 μg/mL) in the presence of rapamycin (2 μM) or Akti-1/2 (1 μM). **(A)** The expression of the glycolysis-related genes *Glut-1, HK2*, and *LDH*α was measured by real-time RT-PCR 24 h after stimulation. **(B)** The Glut-1 expression was detected by flow cytometry and uptake of glucose was measured by detecting MFI of the glucose analog 2-NBDG in CD8+ T cells 48 h after stimulation. **(C)** HK2 expression was detected by western blotting 24 h after stimulation. **(D)** Lactate production was measured by a specific enzyme assay after stimulation for 24 h. **(E,F)** Glycolysis in CD8+ T cells was measured by detecting MFI of Glut-1 and 2-NBDG and lactate production after treating the cells with rapamycin or Akti-1/2 for 48 h. The Glut-1 expression, 2-NBDG uptake, and lactate production in the αCD3+R848 stimulated cells were expressed as fold changes compared to the αCD3 stimulated cells after the corresponding treatment with rapamycin/Akti-1/2. Data are representative of three independent experiments. All data are presented as mean ± SD. The statistical relevance was determined by One-way ANOVA **(A,B,D)** or Two-way ANOVA **(E,F)**: *p < 0.05; **p < 0.01; ***p < 0.001; ns, not significant.

The modulation of metabolic pathways can significantly influence T cell activation and differentiation. TLR-driven early glycolysis reprogramming leads to the activation of DCs (42). It is also known that mTOR-regulated T cell metabolism is required for the initial T cell activation, rapid proliferation, and acquisition of effector functions. Further, mTOR signaling induces complex networks of reprogramming including enhanced aerobic glycolysis to facilitate rapid T cell clonal expansion (41). To investigate TLR7-induced metabolic changes in CD8+ T cells, the expression of Glut-1 and the rate-limiting enzymes HK2 and LDH-α in the glycolytic pathway were determined by real-time RT-PCR (Figure 4A). Increased expression of glycolysis-related genes was found in CD8+ T cells under R848 co-stimulation compared to that with αCD3 stimulation alone. Meanwhile, Glut-1 was also detected by flow cytometry. The uptake of glucose was measured by detecting the mean fluorescence intensity of the glucose analog 2-NBDG. A significantly higher Glut-1 expression level and increased MFI of 2-NBDG in CD8+ T cells were not achieved at 24 h but were detected at 48 h upon αCD3+R848 treatment compared to that with αCD3 alone (Figure 4B, Figure S7D). HK2 expression at the protein level was also significantly increased with αCD3+R848 treatment compared to that with αCD3 alone (Figure 4C). In addition, the production of lactate by aerobic glycolysis was measured in the culture supernatants, showing that R848 could enhance lactate production by CD8+ T cells in the presence of αCD3 (Figure 4D).

We further addressed whether TLR7-induced metabolic changes in CD8+ T cells are dependent on mTOR signaling. Inhibitors of the mTOR signaling pathway were applied to cultures of CD8+ T cells and the mean fluorescence intensity of Glut-1 and 2-NBDG in CD8+ T cells was analyzed. Glut-1 expression and 2-NBDG uptake were significantly increased with αCD3 treatment and further enhanced by R848 at 48 h; however, this was decreased or abolished by rapamycin and Akti-1/2, respectively. Lactate production was also reduced when the mTOR pathway was blocked, even in the presence of αCD3 and R848 (Figure 4E). By analyzing the fold change of the glycolysis-related parameters in the αCD3+R848 stimulated cells compared to the αCD3 only stimulated cells, the R848-induced increase of Glut-1 expression and Lactate production were significantly decreased in the presence of rapamycin and Akti (Figure 4F). Thus, the mTOR signaling pathway plays an important role in mediating the TLR7-induced elevation of the glycolytic metabolism in CD8+ T cells.

### Glycolysis Is Essential for the TLR7-Mediated Enhanced Effector Function of CD8+ T Cells

Glucose is an essential energy supply in cell culture media, and glucose deprivation can lead to impaired T cell activation (43). To confirm that glycolysis drives the activation and functionality of CD8+ T cells, it was blocked with either the glucose analog 2-DG, which inhibits cellular hexokinase or the removal of glucose from culture media. To maintain the energy supply, 1 mM sodium pyruvate and 2 mM L-glutamine were added to the glucose-free medium. Annexin V+7AAD staining was then used to exclude apoptotic cells (Figure S8).

**Figure 5:**
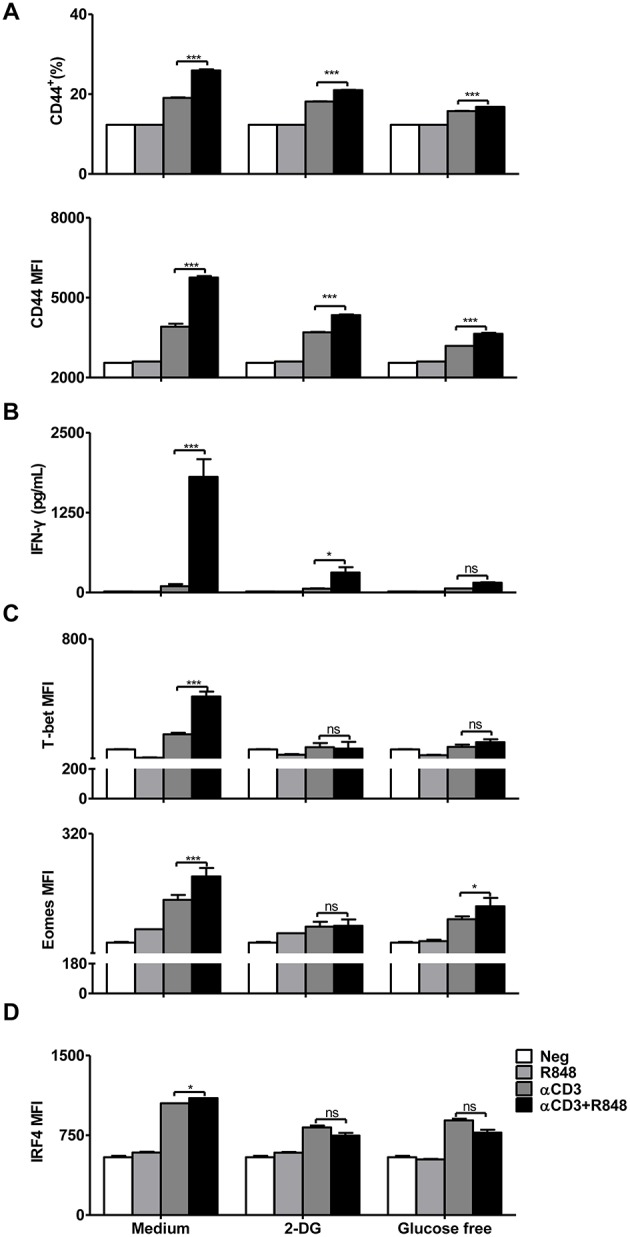
Inhibition of glycolytic metabolism abolishes the functionality of CD8+ T cells. Purified CD8+ T cells were stimulated with αCD3 antibody (5 μg/mL) or/and R848 (10 μg/mL) for 24 h in the presence of 2DG (2 mM) or in the glucose-free medium supplemented with pyruvate. **(A)** The activation of CD8+ T cells was assessed by staining with an αCD44 antibody. **(B)** IFN-γ secretion in CD8+ T cells was determined by a specific ELISA. **(C)** T-bet and Eomes expression in CD8+ T cells was measured by flow cytometry and presented as MFI. **(D)** IRF4 expression in CD8+ T cells was assessed by flow cytometry. Data are representative of three independent experiments. All data are presented as mean ± SD. (*p < 0.05; ***p < 0.001; ns, not significant) Statistical relevance was determined by Two-way ANOVA.

The results showed that blocking glycolysis with 2-DG or glucose deprivation reduced CD44 expression and IFN-γ secretion during CD8+ T cell activation mediated by αCD3 and R848 (Figures 5A,B). At the same time, the expression of the transcription factors T-bet and Eomes was also downregulated in CD8+ T cells (Figure 5C). The expression of IRF4, a transcription factor linked to both metabolism and immunity in CD8+ T cells, was significantly decreased when glucose-free medium was used (Figure 5D).

### Human CD8+ T Cells Respond to the Stimulation of R848 With Enhanced Expression of Activation Markers and Glucose Uptake

Consistent results were obtained using human CD8+ T cells after TLR7 stimulation and blockade of the Akt-mTOR pathway or glycolysis by inhibitors. Human CD8+ T cells were purified by MACS and cultured in plates with bound αCD3 antibody (5 μg/mL) alone or with R848 (1 μg/mL) for 24 h. CD8+ T cells were stained with αCD25 and αCD69 and analyzed by flow cytometry. The uptake of glucose was measured by detecting MFI of the glucose analog 2-NBDG in CD8+ T cells. The stimulation with R848 for enhanced both the expression of activation markers and glucose uptake in αCD3 activated human CD8+ T cells (Figure S9A).

Purified human CD8+ T cells were also stimulated with αCD3 antibody and R848 in the presence of either rapamycin (10 μM), Akti-1/2 (5 μM) or 2-DG (10 mM) for 24 h. The blockade of Akt-mTOR pathway and glycolysis significantly reduced the CD25 expression and glucose uptake in human CD8+ T cells (Figures S9B,C).

### The Transcription Factor IRF4 Plays Role in TLR7-Mediated Enhancement of Glycolysis and Effector Functions in CD8+ T Cells

**Figure 6:**
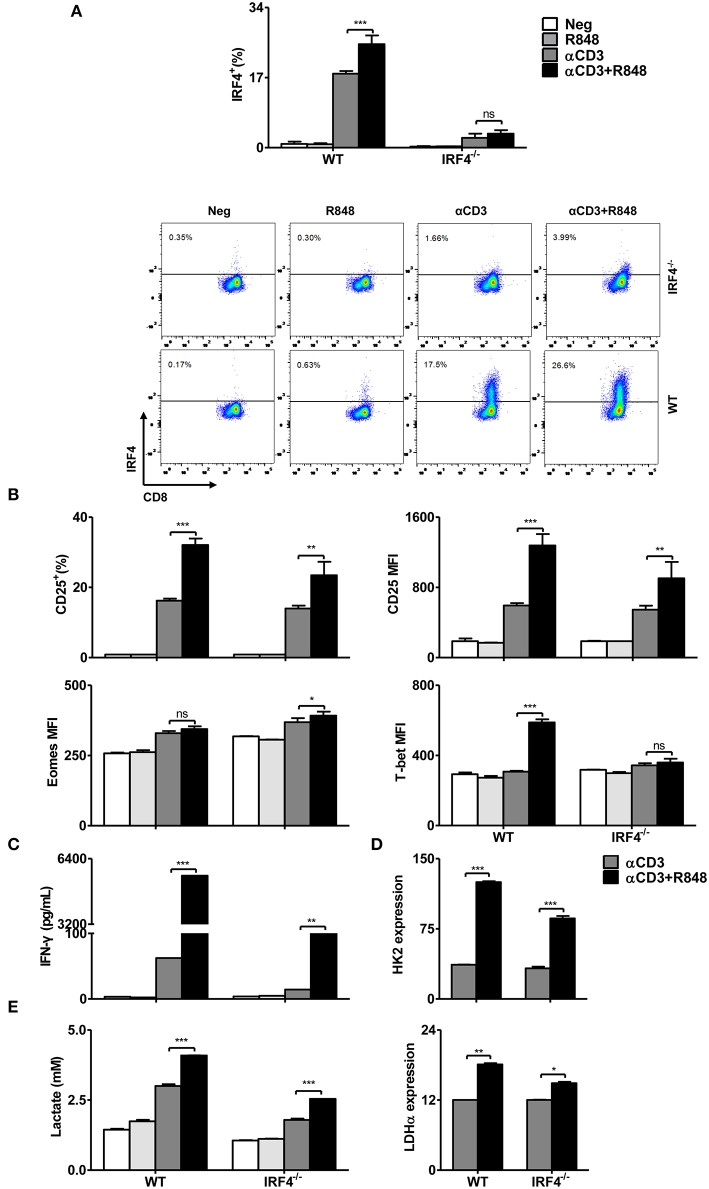
R848-stimulated metabolic and functional changes in CD8+ T cells are partly dependent on the transcription factor IRF4. Purified CD8+ T cells from WT or IRF4^−/−^ mice were stimulated with αCD3 antibody (5 μg/mL) or/and R848 (10 μg/mL) for 24 h. **(A)** Representative dot plots of IRF4 expression in CD8+ T cells as detected by flow cytometry. **(B)** The expression of CD25, T-bet, and Eomes in CD8+ T cells was measured by flow cytometry. **(C)** IFN-γ production by CD8+ T cells from WT and IRF4^−/−^ mice was detected by a specific ELISA. **(D)** The expression of *HK2* and *LDH*α was quantified by real-time RT-PCR. **(E)** Lactate production by CD8+ T cells was measured by a specific enzyme assay. Data are representative of three independent experiments. All data are presented as mean ± SD. The statistical relevance was determined by Two-way ANOVA: *p < 0.05; **p < 0.01; ***p < 0.001; ns, not significant.

IRF4 has been shown to be crucial for TCR affinity-induced metabolic and immune programming in T cells (32). In the presence of TCR signaling, the expression of IRF4 was increased by TLR7 activation, whereas rapamycin and Akti-1/2 treatment could reduce its expression (Figure 5). Thus, we examined the function of IRF4 in R848-stimulated CD8+ T cells derived from WT and IRF4^−/−^ CD8+ T mice, with a focus on its role in remodeling the metabolism. Consistently, IRF4 was not expressed in these CD8+ T cells derived from IRF4^−/−^ mice regardless of the presence of R848 (Figure 6A). Compared to that in WT CD8+ T cells, the enhanced CD25 and T-bet expression mediated by R848 treatment was impaired in IRF4^−/−^ CD8+ T cells, whereas no decrease in Eomes expression was observed (Figure 6B). Moreover, enhanced IFN-γ secretion in R848-stimulated CD8+ T cells was abrogated in the absence of IRF4 (Figure 6C). Thus, R848-mediated augment of CD8+ T cell functions are partially dependent on IRF4 expression.

We next investigated the role of IRF4 in the regulation of cellular metabolic pathways. We measured the expression of glycolysis-related genes by real-time RT-PCR and lactate production using an enzymatic assay. Whereas, HK2 and LDH-α expression and lactate production were increased in both WT and IRF4^−/−^ CD8+ T cells after αCD3 stimulation, R848 treatment led to a further enhancement of these parameters in WT CD8+ T cells, which was markedly diminished in IRF4^−/−^ CD8+ T cells (Figures 6D,E).

BATF, another downstream transcription factor, is also involved in controlling the glycolytic pathway and the regulation of CD8+ T cell functionality. This factor is critical for IRF4-mediated transcription in T cells (44). We thus determined whether the elevated immune function after TLR7 co-stimulation was affected by BATF deficiency using CD8+ T cells isolated from WT and BATF^−/−^ mice. The results demonstrated that the enhanced expression of CD25, CD44, and CD69, production of IFN-γ, and transcription of T-bet and Eomes were reduced in BATF^−/−^ CD8+ T cells (Figures S10A,B).

Thus, IRF4 and BATF are required for the full activation of CD8+ T cells via the regulation of cellular metabolism and effector functions and specifically for the TLR7-mediated signaling to enhance T cell functions.

## Discussion

**Figure 7:**
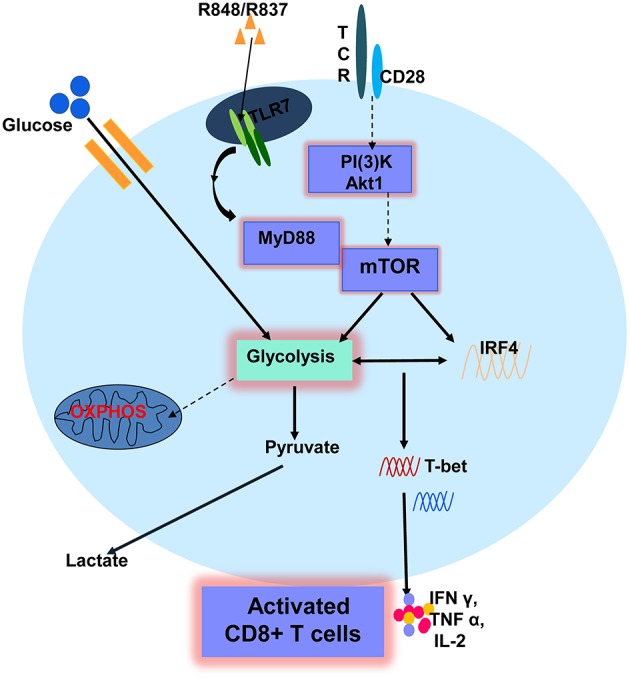
A schematic description of TLR7-mediated enhancement of CD8+ T cell function. TLR7 stimulation promotes the effector functions of CD8+ T cells if CD8+ T cells are primed by αCD3 and express TLR7. TLR7-mediated T cell activation is dependent on MyD88 and AKT-mTOR signaling, leading to the expression of immune-related changes and metabolic alterations in CD8+ T cells, including the upregulation of glucose uptake and glycolysis. Glycolysis was found to be regulated by the AKT-mTOR pathway and a downstream transcription factor IRF4. Glycolysis is critical for TLR7-mediated CD8+ T cell activation and enhanced effector functions of CD8+ T cells. Solid arrows represent signaling pathways identified in this study. Broken arrows indicate potential signaling pathways.

In the present study, we investigated the activation of CD8+ T cells in response to direct stimulation by TLR7 ligands. TLR7 stimulation, specifically by R848, could promote the effector functions of CD8+ T cells *in vitro*. The TLR7-induced activation of CD8+ T cells occurs only if CD8+ T cells are primed by αCD3 and with TLR7 expression and is MyD88-dependent. Furthermore, AKT–mTOR signaling plays a critical role in TLR7-induced T cell activation. While investigating immune-related changes, metabolic alterations in CD8+ T cells, including the upregulation of glucose uptake and glycolysis, occurred after TLR7 stimulation. Glycolysis was also found to be regulated by the AKT–mTOR pathway and the downstream transcription factor IRF4. Blocking glycolysis by either direct glucose deprivation or modulating the mTOR pathway and IRF4 expression was found to impair T cell activation and functions. Our results indicate that TLR7 activation promotes the effector functions of murine CD8+ T cells by enhancing cellular metabolism, and especially glycolysis (Figure 7). Thus, targeting TLR7 and metabolic pathways might represent potential strategies for T-cell-based immune therapies.

T cells that exert optimal effector functions rely on multiple signals, specifically via TCR, co-stimulatory molecules, and pro-inflammatory cytokines (45, 46). TCR engagement can be caused by either non-specific or antigen-specific stimulation. The activation of T cells is restricted by TCR-binding affinities and kinetics (47, 48). Furthermore, co-stimulatory molecules such as CD28 are required for the full activation of T cells by decreasing the threshold of TCR binding affinity (49, 50). In recent studies, TLRs were found to regulate the functions of human CD4+ T cells (51, 52). Our findings demonstrated that TLR7 can also act as a co-stimulatory molecule in CD8+ T cells. First, TLR7 expression is absent or occurs at a very low levels on naïve CD8+ T cells, whereas it was enhanced in the presence of αCD3. R848 co-stimulation significantly improved T cell activation, cytokine production, and the expression of relevant transcription factors such as T-bet and Eomes. These data demonstrate the contribution of TLR7 to induce full activation of CD8+ T cells. Insufficient TCR signaling results in T cell anergy, leading to immune tolerance or immune evasion (53–55). Thus, our findings hint at the potential use of TLR7 co-stimulation to rescue the effector functions of exhausted T cells, for example, during chronic viral infection.

TLRs recognize distinct pathogen-associated molecular patterns and attract adaptor proteins such as TRIF and MyD88 to support the further accumulation of kinase-like IL-1 receptor-associated kinase-4 (IRAK-4) for signal transduction (56). In our study, impaired activation of and cytokine production by CD8+ T cells were observed in MyD88^−/−^ mice, whereas these processes could be further enhanced by R848 in the presence of an αCD3 antibody in TRIF^−/−^ mice. This suggested again that MyD88 regulates T-bet expression, thereby modulating the function of CD8+ T cells.

The TLR–MyD88 pathway has been shown to activate the mTOR pathway, which regulates important cellular processes such as cell survival, metabolism, and autophagy (26, 57, 58). We also found that R848, together with αCD3, could upregulate the expression of mTOR and p-mTOR. Conversely, blocking the AKT–mTOR signaling pathway with Akti-1/2 or rapamycin abolished the enhanced functionality of CD8+ T cells, suggesting that mTOR signaling participates in controlling the function of R848-stimulated CD8+ T cells.

Along with the activation of transcriptional programs, cytokine synthesis and secretion and rapid T cell proliferation and activation require increased metabolic precursors for ATP and biomass synthesis (59). Cellular metabolism was recently found to regulate T cell immunity and could serve as a target for the treatment of several diseases (60). Along with the enhanced functionality and activation of mTOR signaling in CD8+ T cells, our results suggest that TLR7 increases the uptake of glucose, the expression of glycolysis-related genes including *Glut-1, HK2*, and *LDH*α, protein levels of glycolysis-related kinase HK2, and the production of lactate after R848 and αCD3 antibody stimulation. Since mTOR signaling can induce complex networks of reprogramming, including enhanced aerobic glycolysis to facilitate rapid clonal expansion, blocking mTOR signaling with inhibitors also abolished glycolysis in TLR7-activated CD8+ T cells. We are the first to verify that this pathway is responsible for regulating glycolysis in TLR7-stimulated CD8+ T cells. To further clarify the role of glycolysis in TLR7-stimulated CD8+ T cells, glycolytic inhibitors were applied and were found to block the functionality of TLR7-activated CD8+ T cells. Thus, mTOR-regulated glycolysis plays an essential role in the functionality of TLR7-activated CD8+ T cells.

Many transcription factors, such as c-Myc, HIF, IRF4, and BATF, synergistically work downstream of mTOR signaling (30, 41, 61). These transcription factors are involved in the regulation of metabolic and immune reprogramming of CD8+ T cells. While investigating the transcription factors downstream of mTOR signaling, IRF4 was found to be upregulated in CD8+ T cells after R848 stimulation, which occurred in an αCD3-dependent manner. Interestingly, inhibiting mTOR signaling reduced IRF4 expression. Therefore, we compared the function of CD8+ T cells from WT mice and IRF4^−/−^ mice by performing the same treatment. IRF4^−/−^ CD8+ T cells exhibit diminished activation and cytokine production after stimulation with R848, as compared to those in WT CD8+ T cells. In addition, metabolic changes were also decreased, even in R848-co-stimulated IRF4^−/−^ CD8+ T cells, which included a decrease in the expression of glycolysis-related genes and a decrease in lactate production. Thus, IRF4 acts downstream of TLR–MyD88–mTOR signaling and is required for both glycolysis and functional changes in TLR7-activated CD8+ T cells. However, the exact interactions involved in the TLR–MyD88–mTOR pathway, the glycolytic pathway, and IRF4 regulation require further clarification. Given the importance of TLR7 in inducing CD8+ T cell responses in an APC-dependent and independent manner, TLR7 activation might be a promising option for immunotherapeutic approaches to treat chronic infectious diseases and tumors (62).

## Data Availability

The datasets generated for this study are available on request to the corresponding author.

## Ethics Statement

The animal study was reviewed and approved by the experiments using materials from mice were conducted in accordance with the Guide for the Care and Use of Laboratory Animals and were approved by the local Animal Care and Use Committee (Animal Care Center, University of Duisburg-Essen, Essen, Germany and the District Government of Dusseldorf, Germany).

## Author Contributions

QL, EZ, and ML conceived and designed the study and experiments and wrote the paper. QL and EZ performed the experiments. YY, JL, and UD revised the paper. XH, XZ, HX, PL, CK provided the materials and methods. ML supervised this project. All authors have read and approved the final version of this paper.

### Conflict of Interest Statement

The authors declare that the research was conducted in the absence of any commercial or financial relationships that could be construed as a potential conflict of interest.

## Abbreviations

TLRs: Toll-like receptors
HBV: hepatitis B virus
APCs: antigen-presenting cells
αCD3: anti-CD3
TCR: T cell receptor
HIF1α: hypoxia-inducible factor 1α
IRF4: interferon regulatory factor 4
WT: wild type
FV: Friend virus
FV-TCR: Friend virus (DbGagL) T-cell receptor
Glut-1: glucose transporter1
HK2: hexokinase 2
LDH-α: lactate dehydrogenase
R848: resiquimod
R837: Imiquimod
DCs: dendritic cells.
