# Supplementary material for: Toll-Like Receptor 7 Activation Enhances CD8+ T Cell Effector Functions by Promoting Cellular Glycolysis
Source: Front Immunol. 2019 Sep 12;10:2191. doi: 10.3389/fimmu.2019.02191 (PMC6751247; doi:10.3389/fimmu.2019.02191)
Supplement: Supplementary file 1 [file Table_1.DOCX]

Supplementary Material

**Toll-like receptor 7 activation enhances CD8+ T cell effector functions by promoting cellular glycolysis**

Qian Li^1^, Yan Yan^3^, Jia Liu^1,4^, Xuan Huang^5^, Xiaoyong Zhang^5^, Carsten Kirschning^6^; Haifeng C. Xu^7^, Philip A. Lang^7^, Ulf Dittmer^1^, Ejuan Zhang^1,2*^, Mengji Lu^1*^

**^*^Corresponding authors:**

Prof. Dr. Mengji Lu: mengji.lu@uni-due.de

Dr. Ejuan Zhang: zhangejuan@wh.iov.cn

1. **Supplementary Figures**

**Figure S1. TLR7 activation enhances the effector function of CD8+ T cells in mixed splenocyte cultures.**

Splenocytes were isolated from WT mice and stimulated with αCD3 antibody (5 μg/mL) or/and R848 (10 μg/mL) for 24 h. The frequencies of CD44+CD8+ T and CD69+CD8+ T cells were detected by flow cytometry. IFN-γ production in CD8+ T cells was analyzed by intracellular cytokine staining. Data are representative of three independent experiments.

**
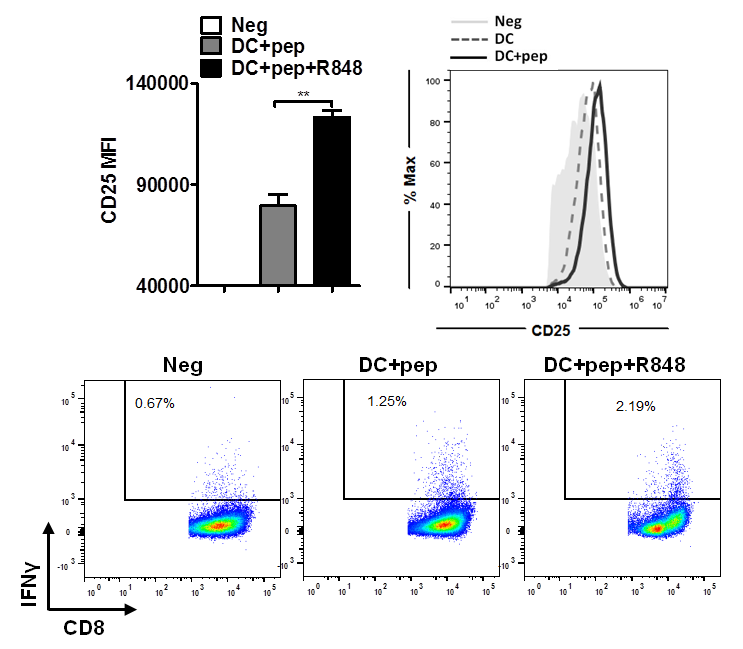
**

**Figure S2. TLR7 activation enhances the effector function of FV-specific CD8+ T cells.**

CD8+ T cells were purified from FV-TCR Tg mice and co-cultured with Friend virus (FV)-peptide pre-coated dendritic cells (DCs) alone or in the presence of R848 (10 μg/mL). Mean fluorescence intensity (MFI) of CD25 and representative dot plots of IFN-γ are shown as determined by flow cytometry. Data are representative of two independent experiments. All data are presented as mean±SD. The statistical relevance was determined by One-way ANOVA: ** p<0.01.

**Figure S3. TLR7 ligands enhance CD8+ T cell activation.**

Purified CD8+ T cells were stimulated in plates with bound αCD3 antibody (5 μg/mL) alone or with R848 (10 μg/mL) for 24 h. The dot plots of CD25, CD44, and CD69 are shown as determined by flow cytometry. Data are representative of three independent experiments.


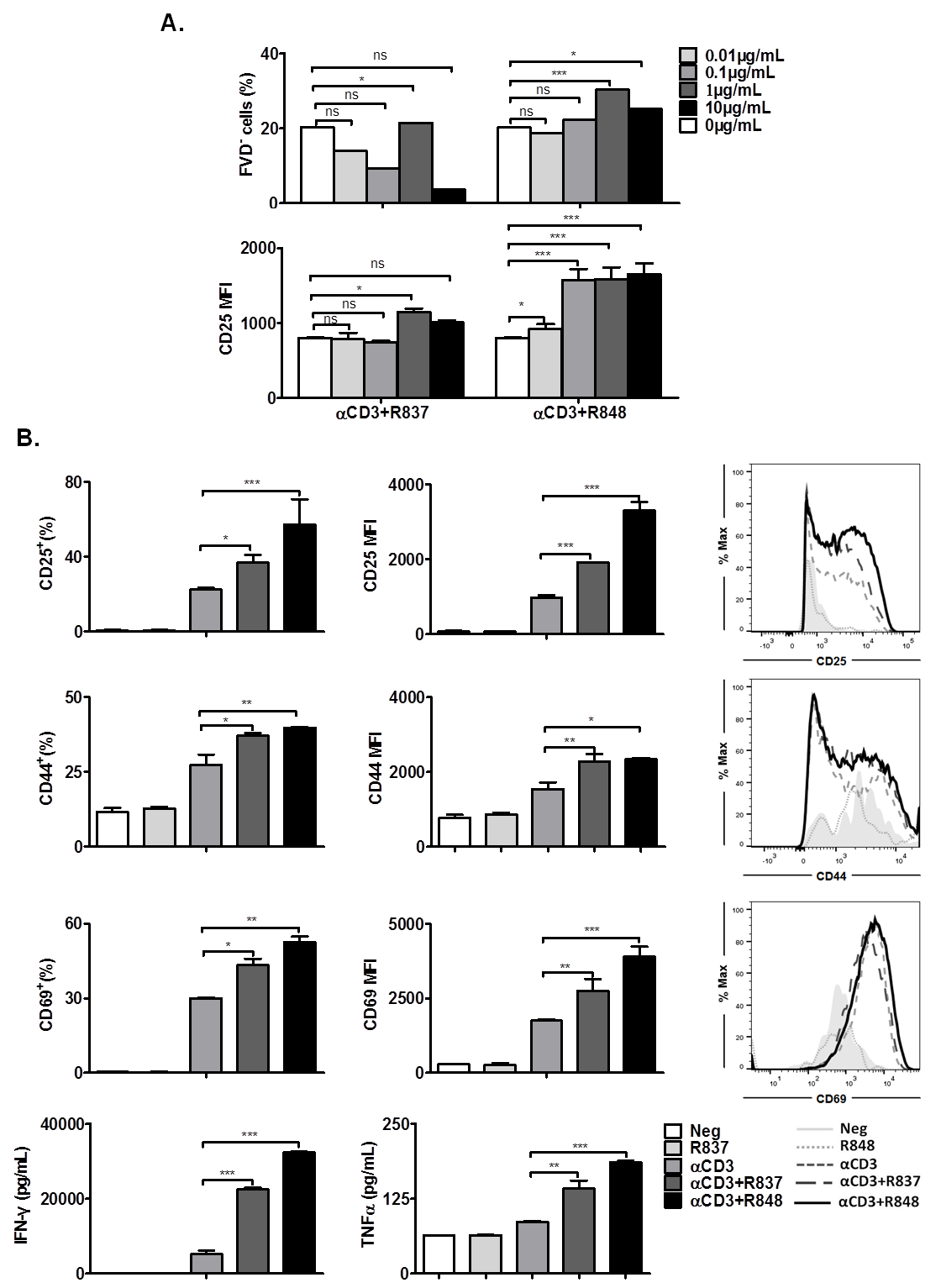


**Figure S4. Comparison of the stimulatory effects of R848 and R837 on CD8+ T cells.**

(A) Purified CD8+ T cells from WT mice were stimulated with αCD3 antibody (5 μg/mL) or/and R837/R848 (0, 0.01, 0.1, 1, and 10 μg/mL) for 48 h. The frequencies of dead cells were determined after staining and exclusion with FVD. (B) Purified CD8+ T cells from WT mice were stimulated with αCD3 antibody (5 μg/mL) or/and R837 (1 μg/mL) or R848 (10 μg/mL) for 48 h. The activation of CD8+ T cells was assessed by staining with αCD25, αCD44, and αCD69 antibodies. IFN-γ and TNF-α secretion by CD8+ T cells were assessed by specific ELISAs. Data are representative of three independent experiments. All data are presented as mean±SD. The statistical relevance was determined by One-way ANOVA: * p<0.05; ** p<0.01;*** p<0.001; ns, not significant.

**Figure S5. Purification of CD8+ T cells by micro-beads and verification by flow cytometry.**

CD8+ T cells were isolated from the spleens of WT mice using Miltenyi micro-beads. The purity of CD8+ T cells was verified by flow cytometry.

**Figure S6. Exclusion of the effect of residual non-CD8+ T cells on CD8+ T cell activation.**

(A) Splenocytes from MyD88^−/−^ mice were labeled with CFSE and mixed with the splenocytes from WT mice at a ratio of 1:1. They were then highly purified using a cocktail antibody in conjunction with bead separation. Purified CD8+ T cells were then stimulated with αCD3 antibody (5 μg/mL) either alone or in the presence of R848 (10 μg/mL) for 24 h. (B) CD69 expression and IFN-γ production in CD8+ T cells were analyzed by flow cytometry. The frequencies and/or MFI of CD69+ and IFN-γ+CD8+ T cells are presented, respectively. All data are presented as mean±SD. The statistical relevance was determined by Two-way ANOVA: * p<0.05; *** p<0.001; ns, not significant.


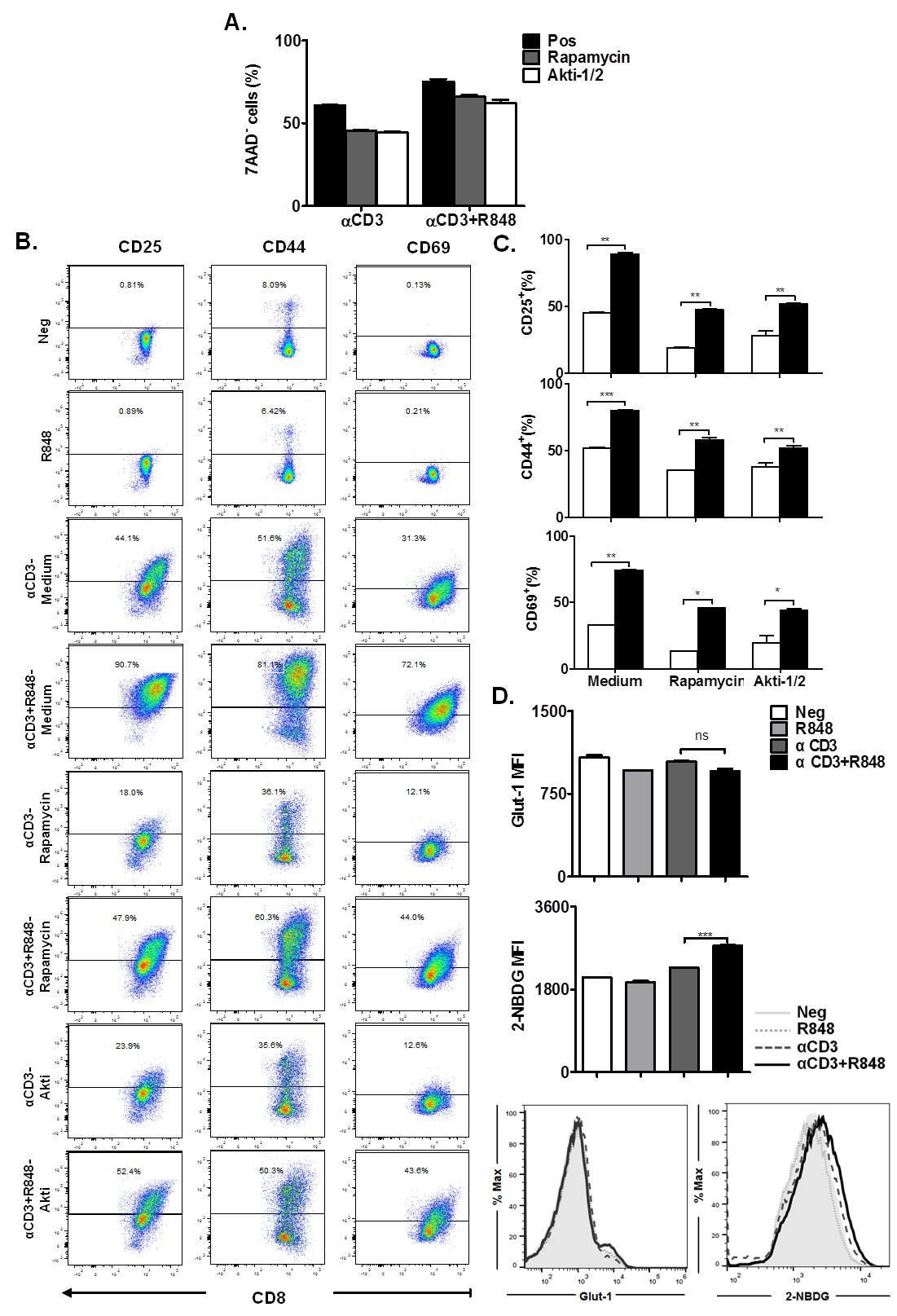


**Figure S7. mTOR signaling regulates the proliferation and activation of CD8+ T cells.**

Purified CD8+ T cells were stimulated with αCD3 antibody (5 μg/mL) with R848 (10 μg/mL) in the presence of either rapamycin (2 μM) or Akti-1/2 (1 μM) for 48 h. (A) Dead cells were labeled with 7AAD. (B, C) The dot plots of CD25+, CD44+, and CD69+CD8+ T cells are shown and analyzed as determined by flow cytometry. (D) The Glut-1 expression was detected by flow cytometry and uptake of glucose was measured by detecting MFI of the glucose analog 2-NBDG in CD8+ T cells 24 h after stimulation. Data are representative of two independent experiments. All data are presented as mean±SD. The statistical relevance was determined by One-way ANOVA (A, C) or Two-way ANOVA (D): * p<0.05; ** p<0.01;*** p<0.001; ns, not significant.

**Figure S8. Apoptosis in CD8+ T cells after treatment with 2DG or cultured in the glucose-free medium.**

Purified CD8+ T cells were stimulated with αCD3 antibody (5 μg/mL) with R848 (10 μg/mL) in the presence of 2DG (1–10 mM) or glucose-deprived medium for 24 h. Apoptotic CD8+ T cells were stained with Annexin V and 7AAD. Dead cells were labeled with 7AAD.


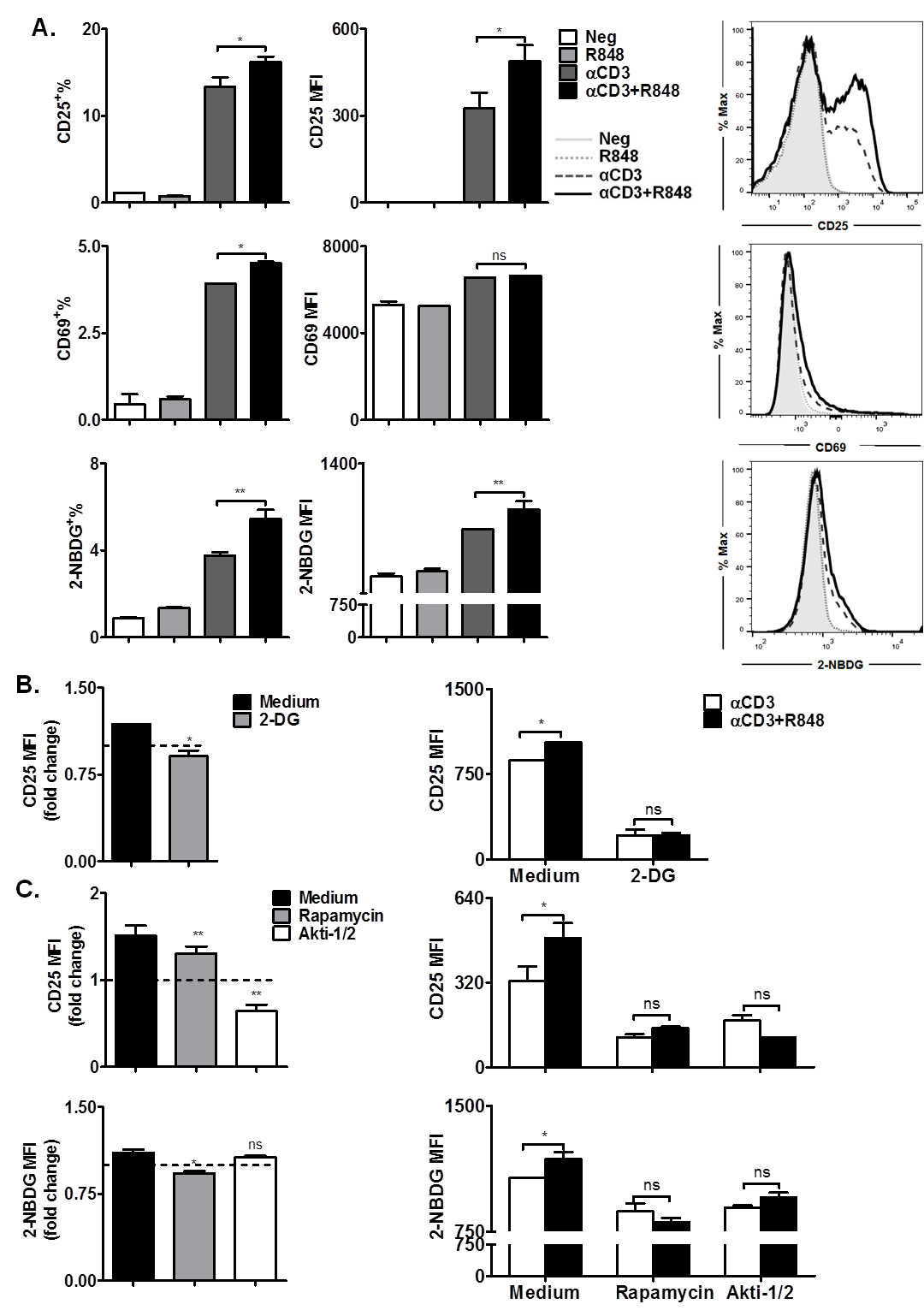


**Figure S9. TLR7 ligands enhance human CD8+ T cell activation and glucose up-taken.**

Purified human CD8+ T cells were stimulated in plates with bound αCD3 antibody (5 μg/mL) alone or with R848 (1 μg/mL) for 24 h in the presence of either 2-DG(10mM), rapamycin (10 μM) or Akti-1/2 (5 μM) for 24 h. (A) CD8+ T cells were stained with αCD25 and αCD69 and detected by flow cytometry. The uptake of glucose was measured by detecting MFI of the glucose analog 2-NBDG in CD8+ T cells. The results are presented as MFI. (B) CD25 expression in CD8+ T cells was assessed by flow cytometry and is presented as MFI. (C) CD25 expression and the uptake of glucose were detected by flow cytometry and are presented as MFI. Fold changes of CD25, and 2-NBDG expression between αCD3+R848 and αCD3 treatment in medium and 2-DG/rapamycin/Akti-1/2 group is indicated by numbers. Data are representative of three independent experiments and presented as mean±SD. (* p<0.05; ** p<0.01; ns, not significant) statistical relevance was determined by One-Way ANOVA in data (A), the fold change graph in (B, C) and Two-way ANOVA in the other graph of (B, C).

**Figure S10. R848-stimulated functional changes in BATF^−/−^ CD8+ T cells.**

Purified CD8+ T cells from WT or BATF^−/−^ mice were stimulated with αCD3 or/and R848 for 24 h. (a) CD8+ T cells were stained with αCD25, αCD44, αCD69, and αIFN-γ and detected by flow cytometry. The results are presented as dot plots and MFI. (b) T-bet and Eomes expression in CD8+ T cells was assessed by flow cytometry and is presented as MFI. Data are representative of three independent experiments. All data are presented as mean±SD. The statistical relevance was determined by Two-way ANOVA: ** p<0.01;*** p<0.001; ns, not significant.

**Figure S11. Gating strategy and fluorescent minus one (FMO) controls for staining.**

FMO controls for each representative staining of antibodies used in flow cytometry panels are displayed.

1. **Supplementary Materials and Methods**

**Mice**

MyD88^−/−^ mice and DbGagL T-cell receptor transgenic (FV-T-cell receptor Tg) mice were bred under speciﬁc pathogen-free conditions at the Institute of Virology of the University Hospital Essen. The FV-T-cell receptor Tg mice were of the C57BL/6 or B6.SJL (CD45.1 congenic) background and 90% of CD8+ T cells contained a TCR specific for the DbGagL Friend virus (FV) epitope (FV-TCR CD8+ T cells).[^1^](#_ENREF_1) BATF^−/−^ mice were bred in the animal facility of Heinrich Heine University, Düsseldorf, Germany. All mice were 6–8 weeks of age. Handling of animals was conducted in accordance with the Guide for the Care and Use of Laboratory Animals and according to the approval by the district government of Düsseldorf, Germany.

T cell culture and activation in vitro

**T cell culture and activation *in vitro***

FV-TCR CD8+ T cells were co-cultured with FV-peptide pre-coated dendritic cells (DCs) and/or R848. For human T cell activation, 96-well ﬂat-bottom tissue culture plates were pre-coated with αCD3 antibody (5.0 μg/mL; eBioscience, Frankfurt, Germany), and human CD8+ T cells were cultured with or without R848 (1 μg/mL; InvivoGen, San Diego, CA, USA) for 24 h (5 × 10^5^ cells/well). For the indicated experiments, human CD8+ T cells were treated with 2-DG (10 mM; Sigma, Germany), rapamycin (10 μM), and Akti-1/2 (5 μM; Sigma, Germany). For all experiments, triplicate wells were performed under each condition (Figure S11).

**Cell staining**

The proportion of viable cells was assessed by 7-AAD (7-Aminoactinomycin D; Germany) or FVD (Fixable Viability Dyes, Germany) exclusion. To analyze apoptosis of CD8+ T cells, cells were stained with Annexin V (Biolegend) and 7-AAD. Fluorescence minus one (FMO) control stains were used to determine background levels of staining (Figure S11).

**Supplemental References**

1. Dittmer U, He H, Messer RJ, Schimmer S, Olbrich AR, Ohlen C, et al. (2004). Functional impairment of CD8(+) T cells by regulatory T cells during persistent retroviral infection. Immunity. 20(3):293-303
